# Malaria and Lyme disease - the largest vector-borne US epidemics in the last 100 years: success and failure of public health

**DOI:** 10.1186/s12889-019-7069-6

**Published:** 2019-06-24

**Authors:** Ilia Rochlin, Dominick V. Ninivaggi, Jorge L. Benach

**Affiliations:** 1 Center for Vector Biology, Rutgers University, 180 Jones Avenue, New Brunswick, NJ 08901 USA; 2 Wetlands and Vector Management, LLC, 22 Rolling Hills Drive, Nesconset, NY 11767 USA; 3 Department of Molecular Genetics and Microbiology, Stony Brook University, Stony Brook, NY 11794 USA

**Keywords:** Mosquito-borne diseases, Tick-borne diseases, Vector control, Public health history, Policy

## Abstract

Malaria and Lyme disease were the largest vector-borne epidemics in recent US history. Malaria, a mosquito-borne disease with intense transmission, had higher morbidity and mortality, whereas Lyme and other tick-borne diseases are more persistent in the environment. The responses to these two epidemics were markedly different. The anti-malaria campaign involved large-scale public works eradicating the disease within two decades. In contrast, Lyme disease control and prevention focused on the individual, advocating personal protection and backyard control, with the disease incidence steeply increasing since 1980s. Control of Lyme and other tick-borne diseases will require a paradigm shift emphasizing measures to reduce tick and host (deer) populations and a substantial R&D effort. These steps will require changing the political climate, perceptions and opinions to generate support among governmental levels and the general public. Such support is essential for providing a real solution to one of the most intractable contemporary public health problems.

## Background

In 1897, Ronald Ross conclusively demonstrated that mosquitoes were not just a “nuisance”, but vectors transmitting the deadly malaria parasite. Walter Reed’s discovery that another feared disease, yellow fever, was vectored by mosquitoes quickly followed. In the nineteenth century, the incidence of these two diseases and other mosquito-borne pathogens in Europe and North America was comparable to that of many tropical regions [1]; by mid-twentieth century, both were eradicated as public health concerns. While mosquito-borne diseases remain largely suppressed, tick-borne diseases are rampant, comprising over 90% of all vector transmitted pathogens in the US and Europe [2–4], with Lyme disease becoming a common infection with estimated 240,000–444,000 annual infections tripling between 2004 and 2016 [3, 5, 6] with much higher most recent estimates [7] The spirochete responsible for Lyme disease was described in 1982 [8]. Almost 40 years post-discovery, its incidence continues to increase, along with other tick-borne diseases such as ehrlichiosis, babesiosis, and anaplasmosis [3].

Why did malaria and other mosquito-borne diseases elicit effective interventions while Lyme and the other tick-borne diseases have not? We examine this question to draw lessons needed to ensure future success against one of the most challenging contemporary public health problems.

### Malaria and Lyme disease in the US

#### Malaria

The malaria parasite is transmitted between humans and *Anopheles* mosquito vectors. The *Anopheles* immature stages inhabit marshy areas, slow moving streams, and margins of still ponds or lakes [9] (Table 1). Temperate malaria transmission is characterized by large fluctuations, low infection rates, and short seasonal longevity; outbreaks occur under increased human-vector contacts. Malaria control began primarily as public works projects founded on entomological knowledge about the mosquito vector [18]. The first report of successful mosquito control appeared in 1902 [19]; the first project targeting primarily malarial mosquitoes in 1910 [14]. Both relied on habitat modifications (drainage and filling) and oiling to kill immature mosquitoes. Although moderate success was attained, those early efforts by private individuals were unsustainable. Realizing that long-term public support was needed, J.B. Smith of New Jersey developed the novel concept of a county-based mosquito control district supported by university affiliated experimental stations [20]. In 1912, the first municipal mosquito control association was created in New Jersey, followed by other states.

**Table 1:** Comparison of Malaria and Lyme diseases epidemics in the US

	Characteristics	Malaria	Lyme Disease
Transmission cycle and biology	Vectors	*Anopheles* mosquitoes	*Ixodes* ticks
	Reservoirs (transmission)	Humans (human to human)	Small rodents (enzootic)
	Pathogens	Protozoa (*Plasmodium* spp)	Bacteria (*Borrelia* spp)
	Vector longevity and pathogen persistence	Short (days – months)	Long (years)
	Vector aggregation (habitat)	Immature (wetlands)	Adults (deer)
Geography	Hyperendemic areas	Deep South	Northeast, upper Mid-West
	# states	13 [10]	14 [11]
	#counties	369 (in 1945) [10]	318 [11]
	Populations at highest risk	Rural	Suburban
Control and prevention	Main control and prevention methods	Habitat modification, biological, insecticides	Personal protection, backyard landscaping, public education
	Spatial scale of control efforts	Very large (state, country)	Very small (personal, backyard)
	Main target of preventative measures	Mosquito vector	Humans
Economics and organization	Dedicated control agency	Local mosquito control district, federal (WPA), private (Rockefeller institute) [12]	None
	Funding (in 2010 $)	$58,278,544 ($6,315,000 in 1948, 3.65% inflation) [10]	$73,620,756 (annual average 2005–10) [13]
	Major expenditure	Personnel, equipment, and supplies for control (> 90%) [10, 14]	Academic and clinical research (87%) [13]
	Jurisdiction over habitat	State and local Public Health Laws, most publically owned	Unclear, habitat mostly privately owned
Statistics and trends	# infections reported/[estimated]	68,289/[278,000-695,000] in 1941 [15]	36,000/[296,000–376,000] annually in 2005–2010 [6]
	US incidence rate	51.8/100,000 (1941) [15]	8.3/100,000 [11]
	Incidence rates in hyperendemic areas	100–400/100,000 [16]	10–90/100,000 [11]
	Peak mortality in the US	> 4/100,000 or ≈ 5000 [17]	Rare
	Trends post discovery	Declining, eradicated by 1950s	Over 3-fold increase since 1990s

The solution to malaria was seen chiefly in vector control and secondarily as the hygienic challenge of human exposure to mosquito bites. These techniques displaced quinine, which was a known prophylactic, but did not lead to actual reduction of the pathogen in the environment [21, 22]. While drainage, ditching, and oiling remained the mainstays of malaria mosquito control, new methods were developed by the 1920s. The mosquitofish (*Gambusia affinis*) was identified as a very efficient predator of mosquito larvae and the backbone of biological control [12, 23]. Paris Green, the first insecticide targeting immature aquatic stages of mosquitoes, was found to be extremely effective in reducing malaria incidence [12, 21, 24]. The county commission efforts quickly brought the desired results. In New Jersey, malaria declined from a high of almost 800 cases in 1914 to being eliminated by 1925 [20]. The last reported outbreak in New York was in 1922 [25].

**Fig. 1:**
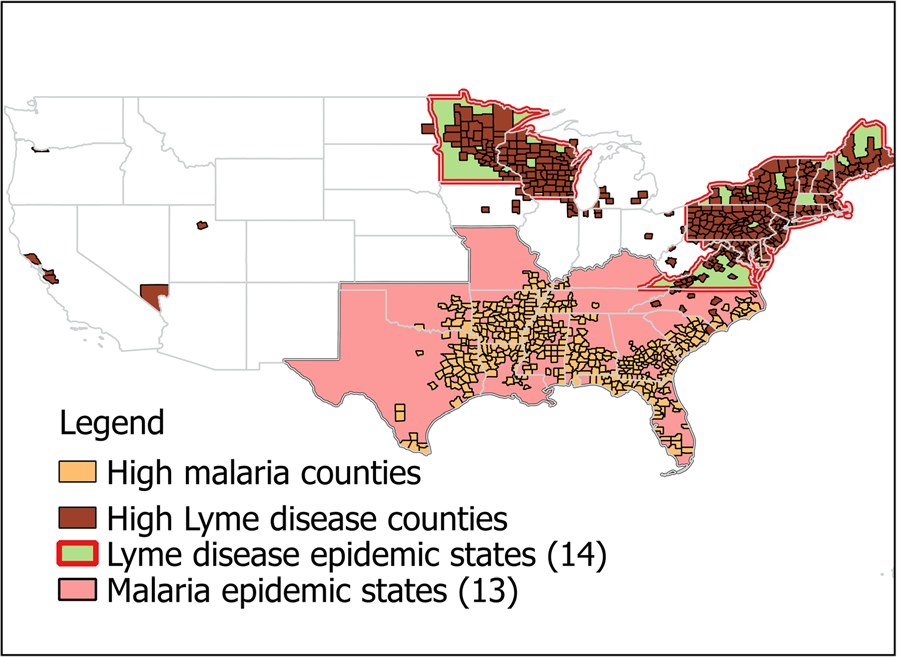
Geographic distribution of hyperendemic areas of Malaria and Lyme disease in the US. Counties with high mortality and morbidity rates of malaria where the National Malaria Eradication Program was carried out in 1945–1949 [10] are in light brown. Malaria hyperendemic states are in shown in pink. High Lyme disease counties are those with > = 10 human cases in 2016 (County level Lyme disease data [26] excluding FL) in dark brown; the hyperendemic states are in green. The map was created by the authors using QGIS open source software. The administrative country level data were obtained from DIVA-GIS free spatial data depository (www.diva-gis.org)

However, malaria persisted in the Deep South (Fig. 1) with morbidity and mortality rates as high as 5402 and 17.4 per 100,000 residents, respectively [16]. The Rockefeller Foundation’s International Health Board initiated mosquito control demonstration projects in Arkansas and Mississippi in 1916–1918 resulting in ~ 70% reduction in malaria index and ~ 90% drop in doctor visits [24]. Encouraged by these successes, 12 southern states assumed mosquito control responsibilities by 1925 [24].

In 1933, the anti-malaria campaign was scaled-up by the federal government through the Civil Works, Works Progress Administration, and Tennessee Valley Authority [27–29]. The scope of these projects was enormous. In 1933, the year of the program’s commencement in 14 southern states, 10,000 km of ditches were excavated draining 120,000 ha and affecting 8 million people [28]. Eventually, about 50,000 km of drainage ditches were created [30]. The 1949 report cited creating 325 reservoirs “to build malaria out”, conducting work over 2.2 million ha affecting 3 million people [10]. These interventions led to steep declines in malaria morbidity from the high of about 150 to 30, and mortality from 45 to 3 (all per 100,000) between the mid-1930s and 1945 [17]. A survey of school aged children found 5.8% positive for malaria in 1932 compared to 0.21% in 1942 [31].

The malaria transmission cycle was suppressed but not eliminated [17, 29]. The National Malaria Eradication Program initiated in 1947 was a cooperative undertaking by 13 southern states (Fig. 1), local health agencies, and the Communicable Disease Center of the U. S. Public Health Service created specifically for the purpose of eradicating malaria [17, 27]. A new strategy of the synthetic insecticide dichlorodiphenyltrichloroethane (DDT) treatments inside residential housing was carried out starting with 245,000 units in 1945 increasing to 1,375,000 in 1948, and eventually to 4,650,000 houses in all 369 counties with excess malaria mortality [10, 17]. Only a small proportion (1–3%) of treated houses harbored *Anopheles* mosquitoes compared to 13–28% of untreated homes [10]. In 1949, the country was declared free of malaria and it was officially certified eradicated 2 years later [27]. As it became clear that DDT and traditional water management methods had unacceptable adverse environmental impacts, newer techniques and materials were developed. The disease was never re-established in the USA despite at least 63 mosquito transmitted malaria outbreaks with 156 cases since 1960s [32].

While malaria was eradicated from the US, another mosquito-borne pathogen, West Nile virus (WNV) has become established since its introduction to New York City in 1999 [33]. Unlike malaria, WNV is a zoonotic disease maintained by the arbovirus circulation between birds and mosquitoes. As such, WNV incidence rate is much lower compared to malaria, however, WNV is much more geographically widespread over North and South America [33, 34].

Control of WNV has presented several significant challenges. WNV outbreaks are unpredictable and diffuse both spatially and temporarily [34]. Action thresholds are difficult to establish, and public health officials are often faced with conflicting public demands for taking action by mosquito treatments and avoiding insecticide use. The economic cost of WNV can be very high exceeding the operational costs of the emergency aerial mosquito control by millions of dollars [35]. However, since mosquito control programs in the US are funded by local authorities [36, 37], these programs may not have the necessary resources, adequate funding, or the political will to implement the most effective actions [36]. Decentralization of vector control efforts from the national to the local level can lead to decreased capacity to deal with mosquito-borne diseases [37], resulting in marked differences in methodology, implementation, and efficacy of mosquito control even among nearby local districts [36].

#### Lyme disease

The reservoir for the causative agent of Lyme disease*, Borrelia burgdorferi*, is the white- footed mouse [38, 39] (see Table 1 for comparison with malaria). Other competent host species, including rodents, rabbits, and raccoons play only a minor role [38, 39]. The blacklegged or deer tick (*Ixodes scapularis*), considered medically unimportant until the 1970s [40] is now recognized as the main vector of Lyme disease [41]. *Ixodes scapularis* has a two- year, three-host species life cycle: larvae feed on small mammals and nymphs attack a very broad range of mammalian and bird species [38]. Nymphal ticks are primarily responsible for Lyme disease transmission to humans and animals [39]. The white-footed mouse is the principal host of immature blacklegged ticks in northeastern US due to its great abundance in the tick’s habitat [42]. Adult blacklegged ticks parasitize large to medium sized mammals [38]. The white-tailed deer is an essential amplifier host [38, 42] responsible for the blacklegged tick’s high population levels, dispersal, and intensified spread in recent decades [39, 43] maintaining *B. burgdorferi* transmission [44–46] and increasing the risk of Lyme disease in humans [47–50]. The increase in Lyme disease and other infections transmitted by blacklegged ticks has paralleled the explosive growth of deer populations [51].

**Fig. 2:**
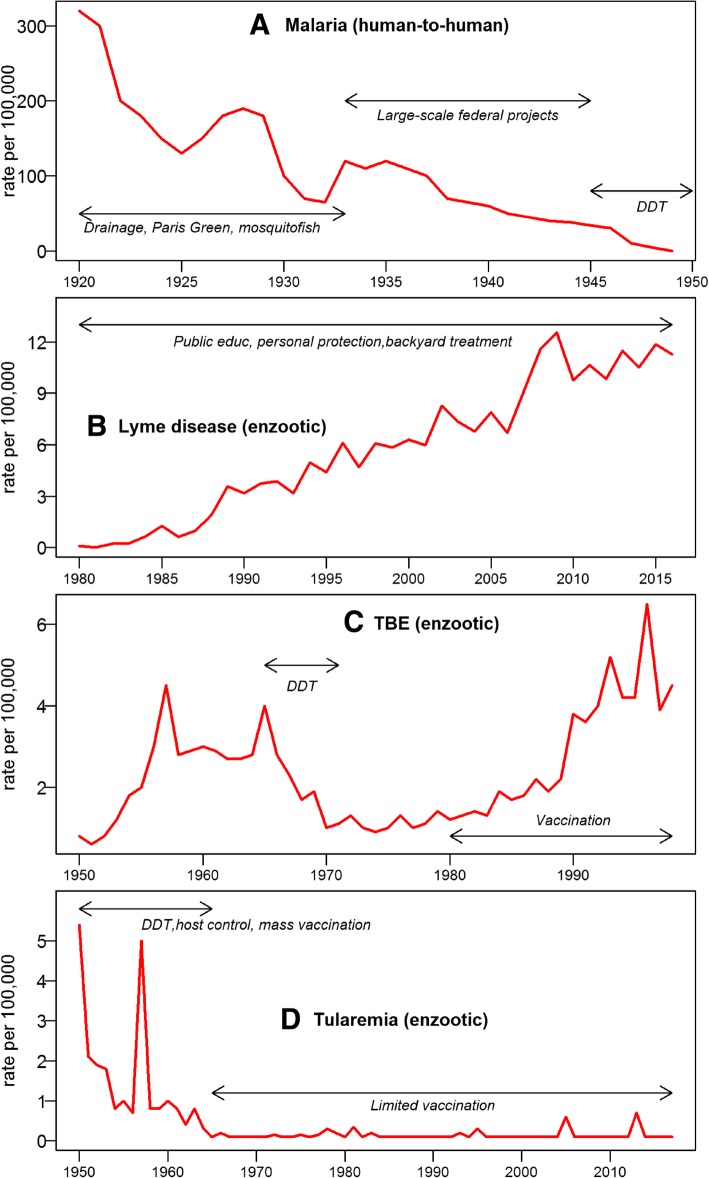
Incidence rates over time (years) for four vector-borne diseases. Arrows and the associated notes indicate main control and prevention measures employed during the time period. **a** Malaria (USA reproduced from [17] human-to-human pathogen transmission by a mosquito vector. Main control measures focused on the mosquito vector and included drainage, ditching, biological control with mosquitofish, and Paris Green larviciding in the early years, large-scale federal public works projects after 1933, and in-house DDT sprays 1945–1949 [10, 12, 21, 29, 30]. **b** Lyme disease (USA, MMWR Morb Mortal Wkly Rep: June 28, 1985 / 34 (10) 376–8; October 06, 1989 / 38 (43) 668–72; Jun 28, 1991 / 40 (10):417–21; March 16, 2001 / 50 (18);181–5; May 7, 2004 / 53 (12) 365–9; June 15, 2007 / 56 (29) 573–6; May 4, 2018 / 67 (12) 496–501) enzootic transmission between tick vectors and rodent hosts. Main preventative measures included public education, personal protection, landscape modification, and backyard treatments [53–56]. **c** Tick-borne encephalitis (USSR/Russia reproduced from [57] enzootic transmission between tick vectors and rodent hosts [57, 58]. Very large-scale DDT applications targeted tick vectors in 1960s to 1971. A TBE vaccine has been widely available since early 1980s. **d** Tularemia (USSR/Russia reproduced from [59] enzootic transmission between mostly tick vectors and rodent hosts, also other routes [59, 60]. Very large scale control efforts in 1940s–1960s included mass-vaccinations, targeting tick vectors with DDT, and greatly reducing the host rodent populations by hunting, trapping, and poisoning. Since 1960s a limited number of people in the risk areas are vaccinated annually

In 1982 the transmission of Lyme disease spirochete to humans by a tick bite was established [8]. Reported Lyme disease cases increased from about 226 in 1981, to 9908 in 1992 (when a uniform case definition was adopted [52]), to over 30,000 in 2016 [26] (Fig. 2b). The number of actual cases are estimated at approximately 300,000 [6, 61], with the reported upper limit of over 400,000 [5] or even higher estimates [7]. The average incidence rate is 8.3/100,000 ranging from about 15 to 83/100,000 in “high incidence” states [11, 26].

No large scale vector control programs have been attempted against ticks transmitting Lyme disease [53]. The Centers for Disease Control and Prevention (CDC) recommends personal protections as the best defense against Lyme disease [54] in addition to landscaping modifications and acaricide applications to individual properties [53–56]. There is very little evidence that any of these measures are effective. Backyard pesticide use reduced the number of ticks, but had no effects on human disease or human-tick encounters [62]. While use of tick repellents increased after educational intervention to about 40%, no differences in exposure to ticks was detected compared to the reference group [63]. Another recommended measure, landscape modifications, did not provide any protection against Lyme disease [64].

Public education is frequently promoted as the key to successful Lyme disease prevention [54, 55, 65]. Educational intervention in a highly endemic Lyme area in Maryland improved knowledge of ticks and Lyme disease and increased use of repellents, but did not decrease exposure to tick bites [63] . This general conclusion has been supported by numerous studies [66–68]. Even the most intensive interventions produced mixed results, e.g. slightly decreasing the rates of self-reporting tick-borne diseases in visitors, but not residents of Nantucket island [69].

These failures of public education to prevent vector-borne diseases agrees with other examples of targeted educational and personal protections interventions, such as elite military personnel acquiring malaria with extremely high incidence rates after short deployments overseas [70, 71]. Failure of these personal protection methods in highly endemic areas is theoretically expected based on modelling [72]. A prerequisite for success is lowering tick population numbers or pathogen prevalence area-wide. The only available area-wide control options to reduce tick populations target hosts for either adult (deer) or the immature (white-footed mice) ticks [73].

Deer populations can be culled directly, or deer can be treated with topical pesticides to kill ticks. Deer population reduction was proposed as the cornerstone of integrated tick management [74]. Drastic reductions or eliminations of deer on islands were accompanied by gradual but largely significant declines in tick populations [45, 75]. Partial deer culls resulted in smaller reductions in tick abundance [47, 76–79]. Significant reductions in tick density and Lyme disease requires very steep deer population changes to empirically estimated 3–5/km^2^ densities [74, 80]. However, these studies leave many open questions with no definitive answer on the link between the reduction in deer populations and human disease.

The topical treatment of deer with pesticides using 4-poster devices resulted in the overall tick reductions of 60–70% [81]. These results were highly variable. Nymphal ticks were always present at the 4-poster study treated areas [81]. Failures of the 4-poster to control tick populations have also been documented [82]. There is some evidence that 4-poster application reduced the rate of tick exposure in treatment areas by approximately threefold, which, however, remained high at erythrema migrants incidence of 137.8/100,000 [79].

Likewise, targeting white-footed mice has a mixed record. Tubes with pesticide-treated nest material generally reduced or eliminated immature ticks parasitizing mice [83], but had no effect the density of questing ticks or their infection rates [84, 85]. Mice bait stations that incorporated pesticide applications reduced the questing tick populations [86]. It is unclear whether these methods provided any human health protection since no human health data were collected.

The rapid rise of Lyme and other tick-borne diseases has occurred despite significant investments by the federal government (Table 1 for comparison with malaria). Between 2006 and 2009, over $100 million dollars on average were spent on tick-borne diseases annually [13]. Over 95% were invested in the academia for microbiological and clinical research supporting the view that research of tick-borne diseases became reductionist, dominated by molecular biology [87]. Vector control and entomological aspects of Lyme disease were largely relegated from the federal public health agencies to the US Department of Agriculture (USDA) [88]. Although much is known about Lyme disease, no clear prescription for prevention has emerged, and the relation of field studies to public health is unknown today, as it was 20 years ago [65].

### Success and failure in vector-borne disease control

The decline of the disease in the human population is the most significant indicator of a public health program’s success. The US anti-malaria campaign was thus tremendously successful, while anti-Lyme efforts are failing (Fig. 2a, b). Malaria consistently declined in 1920–1948 with 50% morbidity reduction every 5 years excepting the Great Depression [17, 28] led by the hyperendemic areas (Fig. 1), where the number of high mortality counties declined from 96 to 14 between 1935 and 1942 [31]. In contrast, the Healthy People 2010 modest goal of 50% reduction in Lyme diseases incidence from 17.4 to 9.7 per 100,000 [65, 89] was not met; instead the incidence had increased threefold.

The underlying biological, technical, and societal causes for these two very different outcomes are complex. Epidemiologically, human to human transmission is more easily interrupted because both the hosts and the vector populations can be targeted to drive the basic reproductive rate (R_0_) below the rate of replacement. For malaria, vector control reduced the number of the pathogen carriers; at the same time, sanitary measures lessened the susceptible human population [90]. Targeting wildlife hosts is much more difficult proposition, which explains why pathogens with wildlife reservoirs continue to persist. This challenge is shared between all zoonotic diseases regardless of vector identity. One of the more well-known examples is West Nile virus, a mosquito-borne zoonotic disease, which remains difficult to control because its wildlife hosts are widely distributed in the environment, and virus amplification is governed by many stochastic processes [33, 34].

Biologically, the difference between mosquitoes and ticks as vectors is important [91]. Daily survival probability is very low for mosquitoes and is high for ticks translating into low population stability but higher transmission potential for mosquitoes compared to an enduring reservoir for infection but lower transmission for ticks [91]. Consequently, tick-borne diseases tend to have lower incidence rates compared to mosquito-borne diseases (Fig. 2). Adult mosquito mobility and intense feeding by immature mosquitoes make them more susceptible to dispersed or ingested insecticides. Aggregated aquatic mosquito stages are easier to target. Ticks inhabit much more cryptic environments, are spread over the suitable habitat, and have only one bloodmeal per each life stage [39, 91]. Tick exposure is mostly peridomestic rather than recreational in the endemic areas of the United States [92, 93]. While recreational exposure on mostly public lands can be more easily mitigated by landscape modification, application of pesticides, and host management [73], residential exposure is more difficult to alleviate due to lack of access, required permissions, highly fragmented habitat, and regulatory impediments.

From the technical perspective, mosquito control was a mature science-based approach with effective techniques and products by the 1930s. Mosquito control personnel could apply chemical larvicides, use predatory killifish for biological control, or make the habitat inhospitable by drainage. No comparably effective tick products or techniques exist and the current methods of targeting the host species and habitat modification have much less impact on the tick populations.

Nevertheless, these challenges cannot fully explain the failure of public health programs since there are historical examples of successful tick-borne disease control. The most instructive cases of tick-borne encephalitis (TBE) and tularemia come from the former Soviet Union (Fig. 2c, d). The anti-TBE campaign focused chiefly on its vector*, Ixodes persulcatus* using DDT applications [58, 94]. The peak of the campaign in 1965–1971 was accompanied by sharp declines in TBE incidence [57] (Fig. 2c). With the cessation of DDT applications in the early 1970s, the incidence of TBE steadily increased remarkably similar to the DDT impact on mosquito populations in North America [95]. The manifold increase in TBE cases in the former Soviet Union and Europe occurred despite existence of highly effective TBE vaccines [96, 97]. Austria, where 85% of the population was vaccinated, was the only country experiencing TBE incidence decline [97]. However, relying solely on a vaccine does not work well in multi-pathogen multi-vector systems [58], and Austria has one of the highest Lyme disease burdens in the world [2, 4].

Arguably, the only long-term success story against a tick-borne disease is tularemia (Fig. 2d) [59, 60]. The enzootic cycle was suppressed by host removal, tick vectors were targeted by DDT applications over vast areas, strict sanitary procedures were implemented, and human protection was conferred by an effective vaccine administered on a mass-scale [60]. As a result of this integrated pest management (IPM) approach tularemia cases fell from a high of approximately 150,000 annually [98] to a few hundred in 1945–1959 stabilizing at < 0.5/100,000 in 1965 (compared to ~ 5.5. in 1950) and maintained by minimal vaccination in the disease hotspots [59]. The effect of DDT was strong in reducing disease incidence, but the resilience of the system seemed to depend on other factors, such as vaccination rate and host removal. More widespread distribution of Lyme disease may present more substantial challenges for these methods.

These examples bring us to the most crucial aspect of vector-borne disease control - the societal dimension - political, administrative, financial, and legal. The importance of socioeconomic changes for the fight against malaria is well documented [99, 100]. In 1943, the National Malaria Society presidential address stated “there is much to learn about malaria, we already have more information than we are using to control the disease…the main obstacles in the way of malaria control today are not so much technical, as they are social” [101], i.e. absence of educated public opinion, inadequate administrative principles, and minimal transfer of knowledge between research and applied control, all of which are valid today.

In the realm of public opinion, Lyme advocacy groups continue to drive public attention toward clinical aspects of the disease and the chronic Lyme controversies [102]. The general public would be hard pressed to find any information on vector control or other real long-term solutions to this problem. Very often novel proposals of dubious nature (such as transgenic mice or ticks) take the front stage, while very little attention is paid to developing public support for the large-scale interventions that will eventually be required to lessen the burden of tick-borne illness.

The same despondent attitude seems to prevail in public health professional organizations. The CDC, a federal agency that was created to fight malaria, doesn’t even have a dedicated tick-borne disease branch despite the fact that the Lyme epidemic is becoming comparable to malaria, at least in the geographic extent if not intensity (Fig. 1). It is hard to envision the public health authorities in the 1930s promoting personal protection as the only way of eliminating malaria. Yet, the organizations tasked with public health protection nowadays do not hesitate to shift the burden to individuals and homeowners. The outcomes are clear when the long-term epidemiological data are compared: while malaria disappeared completely within two decades of vigorous efforts, Lyme disease has been on the rise over the last 40 years (Fig. 2a, b).

The repeatedly heard argument about the lack of resources for tick-borne diseases is refuted by The National Academy reporting funding levels comparable to those during the malaria eradication program (Table 1) [13]. However, 95% out of approximately $100 million annual appropriations are spent on fundamental molecular and clinical research. This imbalance greatly impedes the efforts to find effective control options for tick-borne diseases [87]. These major investments have failed to produce a single useful product for tick control, host management, or a marketable vaccine. The only significant advancement in tick control of the past 40 years, the 4-poster system, was entirely supported by USDA grant [103], and not by the agencies with direct responsibility to protect public health. Contrast this with malaria, where the federal government was directing anti-malaria efforts, providing professional support, labor, development, and eventually delivering a new synthetic insecticide (DDT) to finish the job, with main resources directed toward actual vector control.

Collaboration between the federal government, academia, and local public health agencies on Lyme disease control is very limited in stark contrast to anti-malaria operations staffed, funded, and trained by the US Public Health Service and Rockefeller Foundation [12, 14, 17, 24, 104]. US Public Health scientists were not only conducting surveillance and research studies, but spent a considerable amount of their time supervising fieldwork [14]. Three federal public health stations engaging in applied research and surveillance were established in the hardest hit areas [105]. None of this federal infrastructure has been put in place during the 40 years of the current tick-borne disease epidemic.

In the early part of the twentieth century, environmental protection was not of political concern. Modern tick control methods must not only work, but be environmentally compatible. Tick control has a strong relationship with wildlife management and a new level of interactions with agencies and stakeholders unfamiliar to health authorities. The Lyme disease epidemic is so closely tied to deer overpopulation that control measures will require actively managing and reducing deer populations, at least in heavily populated areas [74].

Realization that control of the vector was essential to anti-malaria efforts engendered the political will and public policies enabling an integrated program of environmental management aimed at that vector to proceed. To emulate this success, the public health agencies, the political system and the public must come to a similar evolution in public policy. First, there must be a realization that tick-borne diseases cannot be controlled without reductions in vector populations. The relationship between ticks and deer must be recognized as the key factor in designing control efforts. At that point, all the involved agencies and stakeholders must come to accept that deer overpopulation must be part of an integrated solution. This becomes even more important as exotic tick species that feed on deer, such as the Asian longhorned tick, are introduced in the US [106]. Research and development (R&D) efforts can then most profitably be directed at innovative methods for reducing deer populations and controlling ticks on deer, where they are most concentrated and vulnerable. This change in paradigm to allow effective control of tick-borne diseases will require strong leadership, especially on the Federal and State levels. Potential steps to improve the coordination among the different levels of government have been outlined [37], with the key component of increasing resources and capacities at the local level where practical vector control is carried out. While changing attitudes and policies with respect to the management of tick and deer populations will take considerable time, it will never happen if not attempted. Meanwhile, R&D funds for tick control need to be greatly increased to reflect the level of threat that exists. The most important change, however, will have to be in the “hearts and minds” of our public health and environmental managers that Lyme and other tick-borne diseases are a problem that demands serious, rationally designed large-scale control measures based on cooperation of all involved parties and significant support by the federal government.

## Conclusions

- Lyme epidemic is comparable to malaria in geographic extent (Fig. 1)
- Lyme epidemic intensity is lower than that of malaria mainly because of its tick vector (Table 1)
- However, Lyme disease incidence is constantly on the rise, while malaria started declining soon after the first targeted intervention in 1910 followed by complete eradication 40 years later (Fig. 2a, b)
- The ecology of Lyme disease, enzootic maintenance, and dispersed tick vectors, contribute to more challenging control compared to malaria with human to human transmission and more concentrated mosquito vectors
- In today’s world, interventions that are controversial or are perceived to have adverse impacts can be successfully opposed, even by relatively small interest groups
- Because Lyme disease is almost never fatal, while there was considerable malaria mortality, the approaches to control these two diseases have been markedly different:
  - ◦ Malaria – larger scale interventions, same as the scale of the epidemic covering millions of acres and millions of people; long-term prevention is carried out by local mosquito control districts
  - ◦ Lyme – a “needle prick” small scale approach, personal protection, public education, backyard treatments
  - ◦ The funding for both epidemics is very similar; however, while the vast majority of malaria funds went for actual control and prevention, the majority of Lyme funds are allocated to academic and clinical research
- There are no historical examples of small scale interventions that successfully controlled a vector-borne or a tick-borne disease
- The successful campaigns against two tick-borne zoonotic diseases (tularemia and TBE, Fig. 2c, d) in the former Soviet Union involved multi-year large-scale sustained measures against vectors and host, and mass-vaccinations
- Based on these examples, success against Lyme epidemic can only be achieved by changing the paradigm:
  - ◦ Large-scale (over the entire epidemic area) and long-term (decades) interventions led by federal and state governments that include measures to reduce populations of vector ticks. It should be noted that tick control would not only reduce the incidence of Lyme disease, but also the incidence of other tick-borne pathogens
  - ◦ The development of more effective, environmentally sound tick control techniques that can be implemented on a wide scale. This will require a substantial R&D effort
  - ◦ Integrated approach of area-wide tick control must be accompanied by very significant deer population reduction and subsequent maintenance at epidemiologically and ecologically acceptable levels
  - ◦ Vaccinations (humans as well as wildlife as a novel method)
  - ◦ Sustained long-term fully funded maintenance efforts by empowering the existing entities such as the mosquito control districts, or by creating new administrative structures such as state authorities
- These measures will require changing the political climate, public perceptions and opinions to generate the required public support

## Data Availability

All data generated or analyzed during this study are included in this published article.

## Abbreviations

CDC: Centers for Disease Control and Prevention
DDT: Dichlorodiphenyltrichloroethane (synthetic insecticide)
IPM: Integrated pest management
R&D: Research and development
TBE: Tick-borne encephalitis
USDA: United States Department of Agriculture
WNV: West Nile virus
